# The unexpected sight: improvement of visual function following intracortical microstimulation of the human occipital cortex

**DOI:** 10.1093/braincomms/fcaf504

**Published:** 2026-02-03

**Authors:** Arantxa Alfaro, Leili Soo, Dorota Waclawczyk, Roberto Morollón, Fabrizio Grani, Eduardo Fernandez

**Affiliations:** Hospital Vega Baja, Servicio Neurología, Orihuela 03314, Spain; CIBER Research Centre on Bioengineering, Biomaterials and Nanomedicine (CIBER BBN), Madrid 28029, Spain; Bioengineering Institute and Cátedra Bidons Egara, University Miguel Hernández, Elche 03202, Spain; Bioengineering Institute and Cátedra Bidons Egara, University Miguel Hernández, Elche 03202, Spain; Bioengineering Institute and Cátedra Bidons Egara, University Miguel Hernández, Elche 03202, Spain; Bioengineering Institute and Cátedra Bidons Egara, University Miguel Hernández, Elche 03202, Spain; CIBER Research Centre on Bioengineering, Biomaterials and Nanomedicine (CIBER BBN), Madrid 28029, Spain; Bioengineering Institute and Cátedra Bidons Egara, University Miguel Hernández, Elche 03202, Spain; John Moran Eye Centre, University of Utah, Salt Lake City 84132, USA; Radboud University, Donders Centre for Neuroscience, Nijmegen, 6525, Netherlands

**Keywords:** electrical stimulation, visual cortex, artificial vision

## Abstract

We describe the case of a participant in a clinical trial investigating intracortical microstimulation of the visual cortex to provide a limited yet functional sense of vision to the profoundly blind. Prior to his formal enrolment, he was completely blind due to bilateral Non-arteritic Anterior Ischemic Optic Neuropathy. Following the initiation of brain electrical microstimulation experiments, he experienced a remarkable recovery of spontaneous vision. The regained sight, after more than 3 years of complete blindness, enabled him to perceive light and motion again and even read large characters and words, enhancing his confidence in mobility and daily activities. We conducted several behavioural and electrophysiological tests to assess and quantify his vision over time.

## Teaser

Alfaro *et al*. report the case of a blind individual who participated in a clinical trial involving intracortical microstimulation of the visual cortex. Following electrical stimulation, the participant experienced a significant recovery of vision, enabling light perception, motion detection and reading of large characters, thus improving mobility and daily activities.

## Graphical Abstract

**Graphical Abstract:**
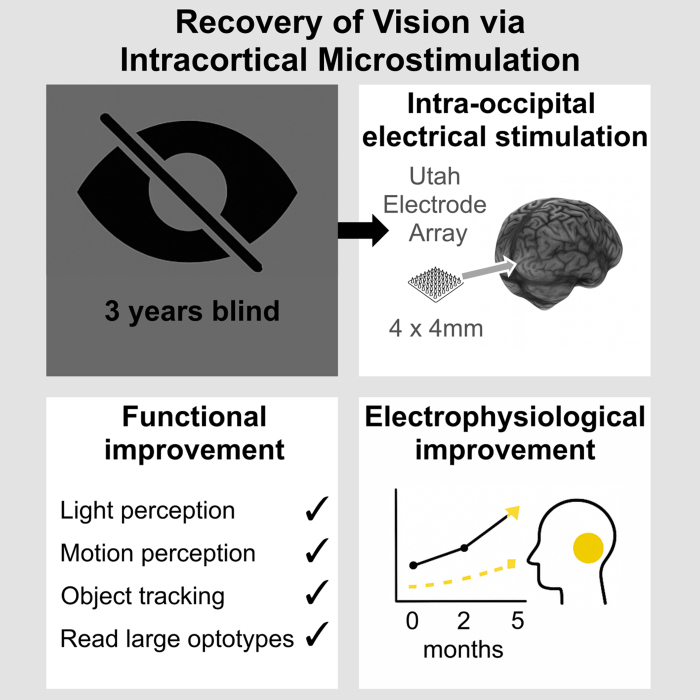

## Introduction

Visual impairment profoundly compromises functional independence and adversely affects physical health, social interactions and emotional well-being. For decades, researchers have sought to encode visual information directly within the visual cortex as a strategy to restore a basic level of vision in individuals who are blind.^1-3^ The pioneering work of Brindley and Lewin^4^ and Dobelle *et al*.^5^ demonstrated that electrically stimulating the visual cortex using implanted surface electrodes could evoke visual percepts (phosphenes), establishing the foundation for this approach. Several years later, Schmidt *et al*.^6^ demonstrated that microstimulation via penetrating microelectrodes could address some limitations of earlier work. These limitations included the need for high currents to evoke percepts and the inability to closely space electrodes; consequently, the perceived phosphenes had low spatial resolution.

Currently, several research groups worldwide are investigating various approaches to apply electrical stimulation to the visual cortex, aiming to restore a limited yet useful visual sense in patients with severe retinal or optic nerve damage.^3,7,8^ However, despite major advances in neuroelectronic interfaces, no cortical visual prosthesis is currently available for clinical use, and further studies are required to realize the clinical goals envisaged for this approach. Key knowledge gaps include understanding the complex neural circuitry underlying visual processing, the optimal stimulation parameters (e.g. frequency, amplitude and waveform) for eliciting specific visual experiences and the long-term neural-tissue responses to chronic stimulation. Furthermore, individual variability in brain structure and function poses significant challenges.

We have suggested that penetrating microelectrode arrays, such as the Utah Electrode Array (UEA), could serve as a foundation for restoring useful functional vision.^8^ We are currently undertaking a clinical trial to evaluate the feasibility of this approach and address some of the remaining challenges. During these studies, a blind participant in this clinical trial experienced a remarkable recovery of vision. Here, we describe this case. Prior to his formal enrolment, he had no light perception. Following the initiation of brain electrical stimulation experiments, he experienced a significant recovery of spontaneous vision, which persisted even after surgical removal of the intracortical microelectrodes.

## Materials and methods

### Study design

The study was conducted under a protocol aimed at assessing the usefulness of a cortical visual prosthesis, based on intracortical microelectrodes, in restoring a limited but functional form of vision in profoundly blind individuals. The protocol was approved by the Ethics Committee of the Hospital General Universitario de Elche and registered at ClinicalTrials.gov (NCT02983370). All procedures and risks were explained in full to the participant and his family, with explicit emphasis on the investigational nature of the work and the absence of expected short- or long-term benefit. The participant acknowledged that the primary aim was to generate knowledge essential for the future development of a cortical visual prosthesis for blind individuals. Written informed consent was obtained in accordance with the Declaration of Helsinki before any study procedures commenced.

### Participant description

The volunteer is a 65-year-old man who was blind due to bilateral Non-arteritic Anterior Ischemic Optic Neuropathy (NAION). He experienced NAION in his right eye at the end of 2018, and 6 weeks later, a repeat episode occurred in the left eye. NAION was diagnosed on the basis of a history of acute, painless, unilateral visual loss with a relative afferent pupillary defect (RAPD), an altitudinal inferior field loss and a hyperaemic and swollen optic nerve with peripapillary splinter haemorrhages. Other potential causes of bilateral sequential optic neuropathy, such as compressive lesions, demyelinating disease, toxic-nutritional states, inherited conditions and systemic inflammatory disorders, were excluded based on clinical features, imaging and laboratory tests. Upon hospital admission, the study of visual evoked potentials (VEP) showed abnormal conduction in both optic nerves and a decreased number of functional axons that was more pronounced at the right optic nerve. His medical history included moderate overweight, hypertension, hyperuricaemia, hypothyroidism and sleep apnoea syndrome (SAS), all well-controlled at his latest clinical follow-up.

Before his formal enrolment in the clinical trial, his visual acuity was no light perception. He had bilateral mydriatic pupils, with no response to light in the right eye and a minimal, barely visible response in the left eye. Funduscopic examination showed pallid optic nerves, consistent with bilateral optic nerve atrophy. Due to the patient’s inability to see the screen and focus on the checkerboards, we were unable to obtain reliable VEP measurements. An extensive systemic and neurological examination was unremarkable.

His cognitive function was intact. He scored 22 on the Montreal Cognitive Assessment (MoCA)—Blind, an adapted version of the original MoCA (scores range from 0 to 22, with a score of 18 or above considered normal). Additionally, he scored 0 on the 10-item Short Portable Mental Status Questionnaire (SPMSQ), where scores range from 0 to 10, with a score of 2 or less considered normal. A detailed psychological assessment revealed no abnormalities. He had good family support and was proficient in using accessible technology for blindness, although he was not a Braille reader.

### Surgical procedures

**Figure 1:**
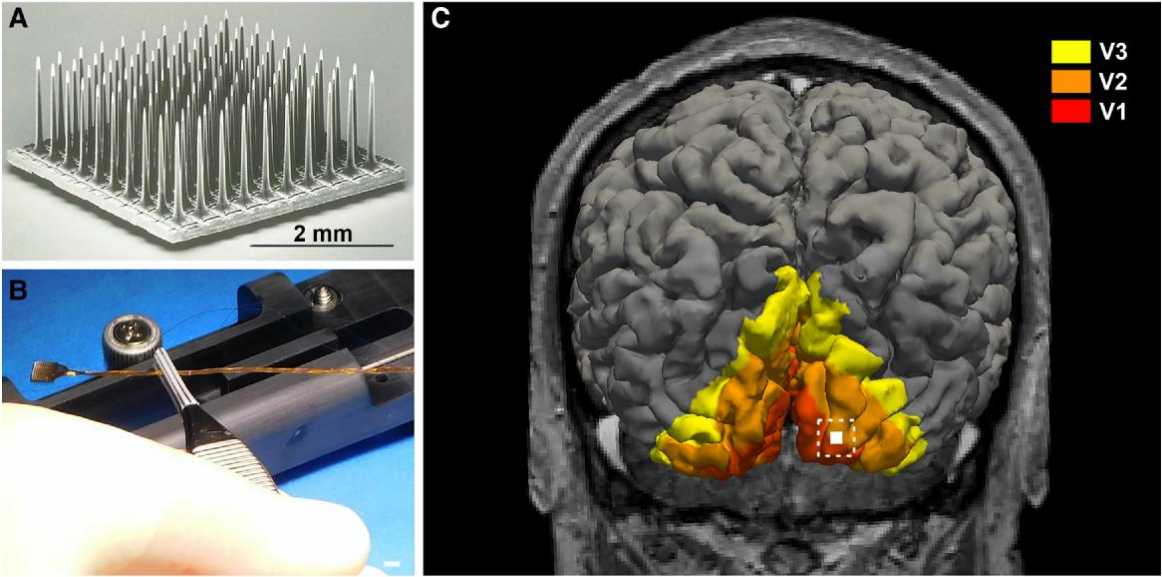
**Implant and retinotopic localization of the Utah Electrode Array (UEA).** (**A**) Scanning electron microscopy image of the UEA. Scale bar, 2 mm. (**B**) Image of the UEA to be implanted during surgery. Scale bar, 2 mm. (**C**) Predicted retinotopic map organization superimposed on the 3D reconstruction of the volunteer’s cerebral cortex with the implantation site indicated.

Three years and 10 months after the bilateral NAION, we surgically implanted a 100-electrode intracortical microelectrode array (Utah Electrode Array, Blackrock Microsystems) at the subject’s occipital pole, near the border between V1 and V2 (see Fig. 1), following previously described procedures.^8,9^ Briefly, following antiseptic scalp preparation, a small craniotomy was performed, centred over the planned implantation site. The implantation site was chosen based on the patient’s cortical anatomy, the reliable elicitation of TMS-evoked phosphenes in that region and the goal of targeting early visual areas (V1/V2) while avoiding major cortical blood vessels. The dura was opened towards the midline to expose the brain surface, and the Utah Electrode Array (UEA) was implanted with a pneumatic inserter (Blackrock Microsystems). The electrode’s external connector was anchored to the skull with six 5 mm titanium microscrews. The final array positioning was recorded through intraoperative photographs and confirmed through postoperative CT imaging that was co-registered with the preoperative 3D MRI.

### Assessment of visual acuity

Assessing vision in subjects with ultra-low vision is a significant challenge. We used the Basic Assessment of Light and Motion (BaLM) and the Freiburg Acuity and Contrast (FrACT) tests to measure visual acuity in this patient.^10,11^ These standardized visual tests include specific assessments for light perception, temporal resolution, light location, motion direction, acuity and contrast sensitivity. Pupils were undilated, and no refractive corrections were used. Measurements were performed on the right eye and left eye separately and then binocularly. During the monocular tests, the non-tested eye was patched.

### Visual evoked potentials (VEPs)

We recorded VEPs following ISCEV standards.^12^ A pattern reversal black and white checkerboard, consisting of 16 by 16 squares with each square covering 4° of visual angle, was presented 100 times at 1 reversal per second on an LG OLED monitor (UltraGear 48GQ900-B) with a 120 Hz refresh rate, positioned 50 cm from the subject. The luminance levels were 77.13 cd/m^2^ for white and 0.16 cd/m^2^ for black (Michelson contrast >99%). In each session, from the two averaged waveforms, we extracted the peak amplitudes and latencies of N1 (a negative peak between 60 and 110 ms after the pattern reversal), P1 (first positive peak after N1) and N2 (first negative peak after P1) components; calculated the peak-to-peak amplitudes for P1 (P1–N1) and N2 (P1–N2); and estimated stimulus-unrelated noise as the peak-to-peak amplitude in the 100 ms pre-stimulus baseline (−100 to 0 ms). To study the dynamic visual processing, we recorded steady-state visual evoked potentials (SSVEP) using electroencephalography (EEG) from 62 scalp electrodes while the participant viewed vertical sinusoidal gratings reversing pattern at 7.5 Hz.^13^

### Visual tasks

To study the state of the subject’s residual vision over time, we employed a series of tasks, each of increasing complexity. These tasks required searching, identifying, distinguishing and/or tracking diverse objects, shapes, letters and numbers. We made it a priority to consistently dedicate at least 30 min each day to these activities. Detailed descriptions and performance analysis are provided in the Supplementary material.

### Statistical analysis

BaLM and FrACT results are reported descriptively. Visual acuity is expressed in Snellen units, and test results are interpreted using standard vision classification categories.

For the analysis of VEPs, we averaged noise estimates across all sessions, reporting the group mean and standard deviation for both eyes separately. Latency (N1, P1, N2) and amplitude (P1–N1, P1–N2) measures at four sessions—pre-surgery (‘before’), 2 months (‘2 months’) and 5 months (‘5 months’) after the start of the clinical trial (trial ended at 6 months) and 6-month follow-up (‘6 month f-u’)—were each compared against a healthy control sample (*n* = 5 controls) using Crawford and Howell’s modified *t*-test.^14^

Two-tailed tests were applied to N1, P1 and N2 latencies and peak-to-peak N2 amplitude (P1–N2). One-tailed tests (hypothesizing a decrease) were applied to peak-to-peak P1 amplitude (P1–N1) due to extensive literature supporting P1 decrease in NAION.^15^ For latencies, *P*-values from the 24 comparisons per outcome (2 eyes × 3 components × 4 sessions) and for amplitudes, *P*-values from the 16 comparisons per outcome (2 eyes × 2 components × 4 sessions) were adjusted using the Benjamini–Hochberg false discovery rate (FDR) procedure,^16^ with significance declared at $p_{FRD}$ <0.05. We also calculated the effect sizes (Cohen’s *d*) and percentage difference from controls. The percentage difference (%Δ) was computed as:

(1) $$\text{\%}\Delta =100\;\times \;\frac{X_{p}-\mu_{control}}{\mu_{control}}$$

where $X_{p}$ is the patient’s value and $\mu_{control}$ is the control mean.

To quantify the observed increase in stimulus-evoked SSVEP amplitude over four recording sessions, we performed within-subject paired *t*-tests on the three highest response occipital cortical channels. Additionally, SSVEP amplitudes were compared against our control sample (*n* =5) using the same Crawford and Howell modified *t*-test applied in the VEP analysis. A detailed statistical report is presented in the Supplementary material.

## Results

Prior to the implant surgery, we attempted to assess the basic perception of light and motion in our blind volunteer using the BaLM test. However, he was unable to see the screen, and the results were below chance level.

Two days after the surgery, while the subject was still in the hospital, we began preliminary experiments to assess phosphene thresholds on some individual electrodes that reliably recorded multiunit activity. For these experiments, we used stimulus trains of 50 cathodic-first, charge-balanced biphasic pulses, with different intensities, a phase duration of 170 μs/phase and a frequency of 300 Hz.^8^ During these studies, the subject suddenly reported beginning to perceive some lights and movements in front of him. Since we were aware of the possible increase of spontaneous positive visual phenomena in the first weeks after the implantation of penetrating microelectrodes in the human visual cortex,^8^ we explained this to the subject and attempted to reassure him. However, he persisted in asserting that he could now discern the movement of some researchers near him. His wife was present in the room at the time and confirmed that this was the very first time he had reported seeing after his blindness. Then, one of the researchers began moving their arms in various directions in front of the subject, asking him to describe their position. The subject was able to accurately describe the researcher’s arm position every time. He reported perceiving something akin to a shadow, along with the movement of the arms (see Supplementary Video 1).

**Figure 2:**
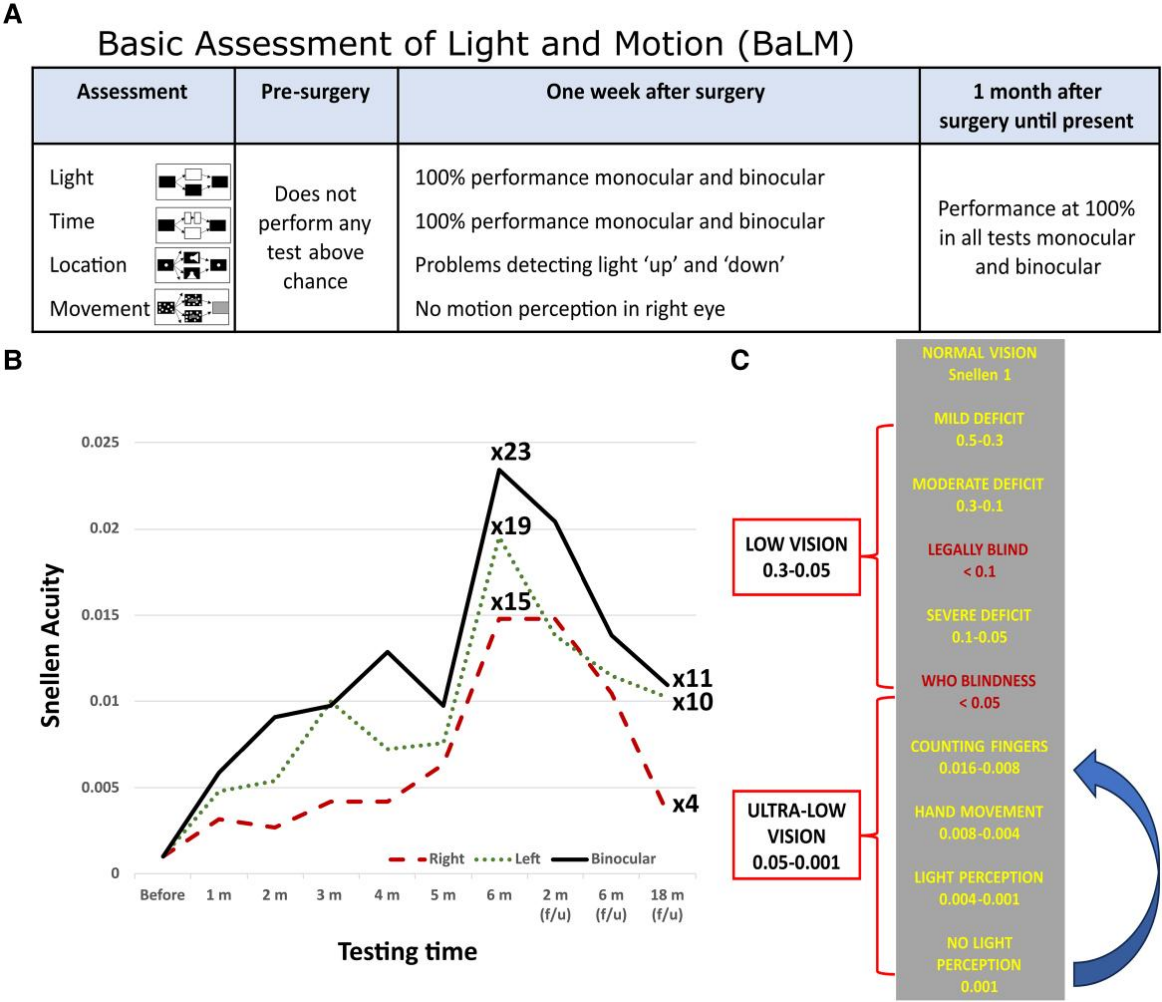
**Results of the behavioural assessments.** All assessments were performed with the microelectrode array switched off, under three viewing conditions: binocular, left eye only and right eye only. (**A**) The results of the Basic Assessment of Light and Motion (BaLM Test), used to assess visual function quantitatively in the very low vision range. (**B**) The results of visual acuity (Freiburg test) for the experimental months and follow-up sessions. From the baseline (pre-surgical), there was an increase in visual acuity that was maintained until explantation, followed by a progressive decrease afterward, without returning to pre-implantation levels. This represented a vision gain, with maximum improvements of 23 times the baseline for both eyes, 19 times for the left eye and 15 times for the right eye. (**C**) Classification of vision. Based on his visual acuity, the patient belongs to the ‘ultra-low vision’ category, which corresponds to the blindness range defined by the World Health Organization (WHO). Time-line progress showed a significant improvement in vision from light perception at the time of surgery to counting fingers at explantation and later.

After the onset of his residual vision, we repeated the BaLM test. The results were above the chance rate (Fig. 2A), with ceiling performance in the assessment of light perception 1 week after surgery. We then continued with the experimental plan, which involved electrical stimulation and recording experiments conducted 5 days a week for 3–4 h per day. The BaLM test was repeated once a month throughout the entire experimental period, as well as 2 and 6 months after the conclusion of the experiments during follow-up sessions. The participant performed at ceiling levels in all modules (light perception, temporal resolution, light location and motion direction) by the first month after implantation, and these results were maintained in all subsequent testing sessions, both in binocular and monocular viewing in both eyes (see Supplementary Video 2).

Since the participant was unable to see the screen or perceive any light in the indoor environment, the FrACT test was not attempted before the surgery. However, following the onset of residual vision, he was able to complete the FrACT test. We repeated this test monthly and observed a significant increase in visual acuity over time (Fig. 2B). Six months after the implantation and the start of electrical stimulation experiments, the results of the FrACT test indicated a vision improvement of 23-fold, 19-fold and 15-fold over baseline in binocular, left eye and right eye assessments, respectively. Two months after the explantation of the intracortical microelectrodes, the improvement in vision remained relatively stable. Subsequently, there was a decline over time, particularly in the right eye. However, 18 months after explantation, binocular vision remained 11 times better than pre-implantation levels (see Fig. 2B).

**Figure 3:**
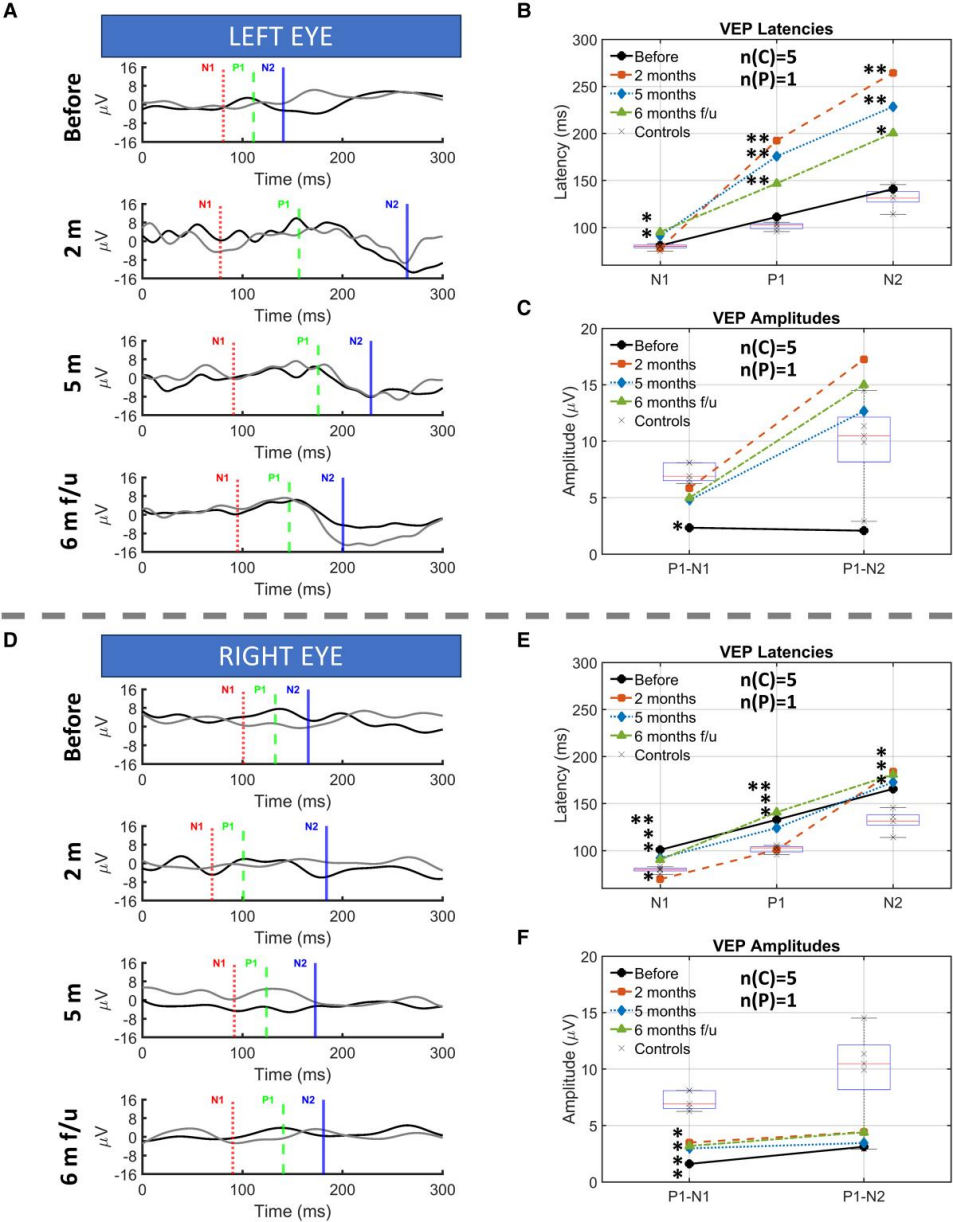
**Comparison of visual evoked potentials between controls and patients.** In all panels, ‘before’ refers to data collected two weeks prior to surgery, ‘2 m’ and ‘5 m’ represents data collected 2 and 5 months after surgery during the experimentation phase, and ‘6 m f-u’ indicates data collected 6 months post-implant removal in a follow-up session, after the experimentation was completed. **(A and D)** Grand-average VEP waveforms for the patient’s left eye **(A)** and right eye **(D)**. Vertical lines mark component peaks: N1 (first negative), P1 (first positive) and N2 (second negative). **(B, C, E, F)** VEP peak latencies (left eye, **B**; right eye, **E**) and peak-to-peak amplitudes (left eye, **C**; right eye, **F**). Boxplots represent five healthy controls (*n* = 5), with individual datapoints presented as crosses. Individual patient values (*n* = 1) are overlaid for each session. To compare patients versus controls, we used Crawford and Howell’s modified *t*-test.^14^ Benjamini–Hochberg FDR-adjusted *P*-values indicate significant group differences in the figure (*P < 0.05; **P < 0.01). Full statistics are provided in Supplementary Tables 1 and 2.

**Figure 4:**
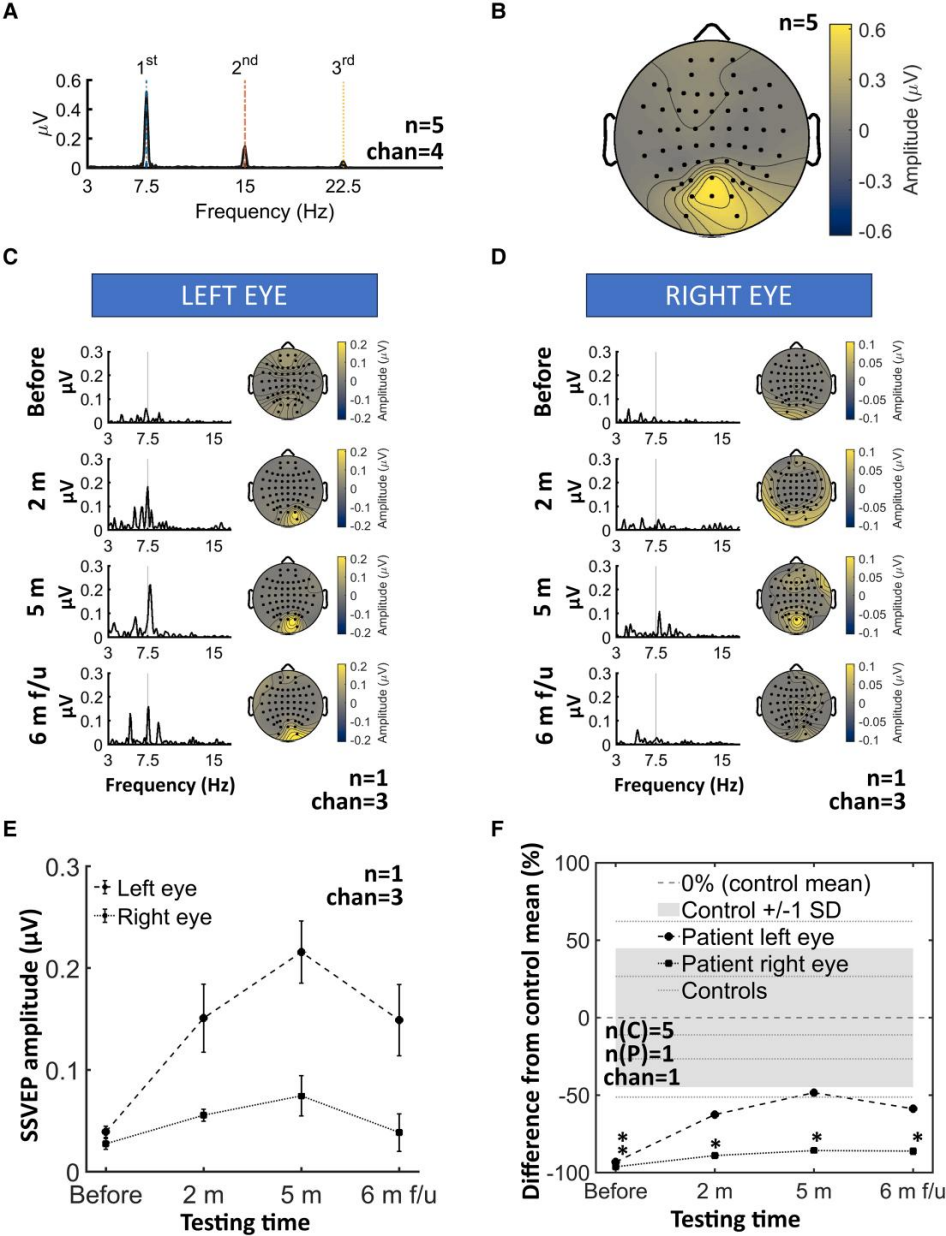
**Steady-state visual evoked potentials in controls and patients. (A)** Grand-average amplitude spectrum across four highest response channels (Oz, POz, O1, O2) from five healthy controls (*n* = 5), showing peaks at the fundamental (7.5 Hz), second harmonic (15 Hz) and third harmonic (22.5 Hz). All health controls plotted separately in Supplementary Fig. 5. **(B)** Scalp topography of the group mean amplitude at the fundamental stimulation frequency (7.5 Hz; colour bar in µV). **(C)** Patient’s left eye amplitude spectra (left panels), averaged across three highest response channels (Oz, O2, CB2) and corresponding topographical maps (right panels) at baseline (‘before’) and at 2 months (‘2 m’), 5 months (‘5 m’) and 6-month follow-up (‘6 m f-u’). **(D)** Same as in (**C**) for the patient’s right eye. **(E)** SSVEP amplitude averaged across the three channels exhibiting the highest response (Oz, O2, CB2) in the patient’s left and right eyes; error bars indicate ±SEM across those three electrode sites. All sessions were compared to baseline (‘before’) using paired-samples *t*-tests, full statistics are provided in Supplementary Table 3. **(F)** Per cent difference of the patient’s SSVEP amplitude at Oz relative to the control-group mean; the shaded grey band denotes ±1 SD of controls and the dashed horizontal line marks 0% (control mean), and the dotted lines represent five healthy controls. Statistical comparisons used Crawford and Howell’s modified *t*-test^14^ to contrast the single patient against controls. Benjamini–Hochberg FDR-adjusted *P*-values indicate significant group differences in the figure (*P < 0.05). Full statistics are provided in Supplementary Table 4.

Prior to surgery, visual evoked potentials (VEPs) were nearly undetectable in both eyes (Fig. 3A and D). After beginning brain electrical stimulation experiments, we observed several negative and positive waves with clear occipital responses compared to the pre-stimulus baseline. Across testing sessions, the right-eye noise floor (peak-to-peak) averaged 2.09 ± 0.75 µV, and the left eye noise floor averaged 1.29 ± 0.54 µV. VEP amplitudes exceeded their respective noise floors in every session except the pre-surgery right eye recording. At baseline, the patient’s visual evoked potentials (VEPs) were temporally normal but exhibited dramatically reduced amplitudes (Fig. 3). When tested at 2 and 5 months after the start of the study, left-eye P1 amplitudes increased while all three VEP latencies (N1, P1, N2) became significantly delayed. In contrast, the right eye remained persistently attenuated in both amplitude and timing. SSVEPs mirrored this pattern: left-eye amplitudes showed transient increases, whereas right-eye responses stayed significantly below control levels (Fig. 4). By the 6-month follow-up, left eye latencies and inter-peak intervals began normalizing alongside stable amplitudes.

Overall, the volunteer exhibited a notable enhancement in his visual acuity and a marked improvement in the performance of daily activities compared to the pre-implantation baseline. He was able to consistently identify shapes and letters (see Supplementary Video 3), and the smoothness of his voluntary movements for grasping objects increased significantly. Additionally, he gained confidence in mobility. He reported that his regained vision was sufficient to boost his confidence in moving around and improve his ability to perform daily tasks, allowing him to gain more independence.

## Discussion

We describe the case of a blind individual with bilateral NAION who experienced a notable recovery of vision after we began electrical stimulation experiments to explore the potential of a cortical visual prosthesis. This recovery allowed him to perceive light, motion and even read large characters. Although our results are based on a single case, they could contribute to the development of new therapeutic approaches and rehabilitation paradigms for individuals with damage to the optic nerve in the future.

While this patient participated in a clinical trial investigating the safety and feasibility of intracortical electrical stimulation as a basis for a cortical visual neuroprosthesis, the stimulation protocol was designed to elicit artificial visual percepts and was not intended to restore or enhance residual vision. Among the four participants tested so far, this patient was the only one who experienced a measurable and sustained improvement in natural visual acuity, suggesting that unique patient-specific factors may have contributed to this outcome. Compared to the other three participants in the trial, this patient retained a minimal pupillary response at the start of the trial in one eye (left eye), whereas the others had no pupillary reflexes. Hence, visual recovery may require the preservation of a minimal structural substrate^17^ and a critical mass of functionally viable neurons.^18^

The adult visual cortex has a certain degree of plasticity,^19-21^ and the present case shows that significant improvement in visual function is possible even after many months of blindness. We recognize that spontaneous improvement in visual acuity is not uncommon among patients following NAION episodes.^22^ Reports indicate that at least three Snellen acuity lines have been regained in up to 42.7% of patients. However, such recovery typically occurs within the first few weeks and rarely extends beyond 2–3 months after the initial damage.^23^ Consequently, the restoration of some visual functions in our blind volunteer after more than three years of blindness is exceptionally unusual.

While it is possible that a small number of residual ganglion cells (RGCs) survive ischaemic injury, their numbers decline significantly over time due to retrograde degeneration.^24^ This suggests that the mechanisms driving recovery in our subject may involve other structures along the visual pathways.^25^ On the other hand, given the retinotopic organization of the visual cortex, improvements in visual function following cortical stimulation are expected to be localized to specific regions of the visual field. Future studies could employ quadrant- or hemifield-specific VEPs or SSVEPs to systematically assess whether such changes correspond to the stimulation site. However, it is important to note that fixation instability is common in individuals with low vision,^26^ which may limit the feasibility of spatially resolved assessments using these methods.

How brain activity influences functional reconnection of the eye with the brain remains poorly understood. The implantation of the UEA and the electrical microstimulation of the visual cortex could have induced neurotrophic-like effects, leading to functional reorganization for processing visual information, as has been reported with retinal prostheses.^27^ Thus, chronic intracortical microstimulation may initiate molecular and cellular cascades analogous to those triggered by endogenous neurotrophic factors, promoting the release of growth-promoting signals, synaptic sprouting and sustained neuronal health.^28,29^ In support of this idea, studies in animal models have demonstrated that postsynaptic neuronal activity may play a critical role in promoting the regeneration of retinal axons.^30^ Furthermore, previous studies in humans using non-invasive techniques such as transcranial electric stimulation (TES) or transcranial magnetic stimulation (TMS) suggest that these approaches can modulate neurotransmitter balance and may offer promising therapies for preserving or restoring vision in various retinal and optic nerve diseases.^31,32^

We also believe that visual training and the motivation of the subject have played a significant role in the partial recovery of some residual vision in this subject. At present, training is the most commonly used strategy to modulate visual plasticity and support visual recovery.^32^ Therefore, the combined effects of intensive, attention-driven visual training^33,34^ and electrical microstimulation could contribute to enhancing function in partially injured pathways that project, directly or indirectly, to higher-order cortex. This approach may unmask latent synapses in early visual areas, reduce local intracortical inhibition^35,36^ and reopen a window of adult visual cortical plasticity,^21,37^ thereby supporting structural and functional reorganization that exceeds the limits of spontaneous recovery alone.

Even considering all these aspects, we should be aware that this unusual visual recovery might vary significantly depending on the visual pathology, residual vision and duration of blindness. Future research will help determine whether this represents an isolated event and will contribute to a deeper understanding of the underlying neurological mechanisms.

## Supplementary Material

fcaf504_Supplementary_Data

## Data Availability

Data supporting this study are available upon reasonable request from the authors. All code created and used in this study is available in the Open Science Framework (OSF) repository and can be accessed at https://osf.io/urxqk/.
