# Supplementary material for: The unexpected sight: improvement of visual function following intracortical microstimulation of the human occipital cortex
Source: Brain Commun. 2026 Feb 3;8(1):fcaf504. doi: 10.1093/braincomms/fcaf504 (PMC12957922; doi:10.1093/braincomms/fcaf504)
Supplement: fcaf504_Supplementary_Data [file fcaf504_Supplementary_Data.zip › Supplementary_material.pdf]

<u>Supplementary material</u>

Visual tasks

Overview

Visual tasks were added to the research protocol in response to the improvement in residual vision to assist the participant use and potentially improve their recovered vision. We implemented a multifaceted approach to vision rehabilitation, gradually increasing demands on performance. We employed three distinct categories of tasks, beginning with computerized tasks that required identifying, distinguishing, searching and/or tracking of different objects, shapes, letters, and numbers. Secondly, to mimic real-world scenarios, we implemented Activities of Daily Living (ADL) tasks. As the last part of the visual tasks, we trained Orientation and Mobility (O&M) within our laboratory environment (StreetLab and Apartment) and on a Virtual Reality (VR) treadmill. All behavioural tasks were informed by prior work in simulated prosthetic vision [1-5] and by studies conducted with retinal implant patients [2,6-9].

Methods and results

Screen-based tasks

For the screen-based tasks, we used an LG OLED monitor (UltraGear 48GQ900-B) with a 120 Hz refresh rate, positioned 50 cm from the subject. Stimuli were shown either as white on a black background or black on a white background. Although neither configuration yielded a clear performance advantage, maximal contrast was essential for reliable task performance. All testing was conducted in a darkened room with the participant's head stabilised using a chin rest.

Shape localization, recognition and tracking

We presented different objects on a computer screen for localization and identification (Supplementary Figure 1A). The patient was instructed to report the shape of the object, point to its centre and track the edges with his finger. Shapes were either presented in isolation or as groups of two or three (Supplementary Figure 1B), always in a random location of the screen. Additionally, we presented high contrast versions of everyday objects on a computer screen (such as a fork, plate, knife, spoon, scissors, cup etc.) and had the same physical object ready on the table for visual and tactile inspection (Supplementary Figure 1C).

The patient had no difficulty in reporting simple high contrast shapes; however, he was not able to report the objects that were more complex or without high-contrast edges. In different trials, he would need more than 5 seconds to locate the shape but in general, he was always successful. The centre pointed out was always slightly off to the north-east side of the object (as indicated in Supplementary Figure 1D). Subjectively, he noted the greatest visual improvement in his lower-left central field—contralateral to the implant. Because he habitually fixated on the upper-right corner of each target to recognize it, this eccentric fixation likely accounts for the systematic north-east pointing error.

Letter and number recognition

To complement FrACT, we presented single letters of the entire alphabet and numbers (1-9) in various sizes in the centre of the screen. The sizes of the letters increased when the response was incorrect and decreased when the response was correct. Patient was able to identify letters and numbers in similar sizes expected from the results of the FrACT with a decreasing reaction time (Supplementary Figure 1E). Additionally, after 5 months of training he was able to identify letters presented only for 500 ms binocularly and with left eye. With the right eye, significant visual inspection with eye movements was necessary to identify the letters. We also presented short two to three letter Spanish words (the patient's native language), which he was able to read after thorough visual inspection, sometimes lasting up to 10-20 seconds.

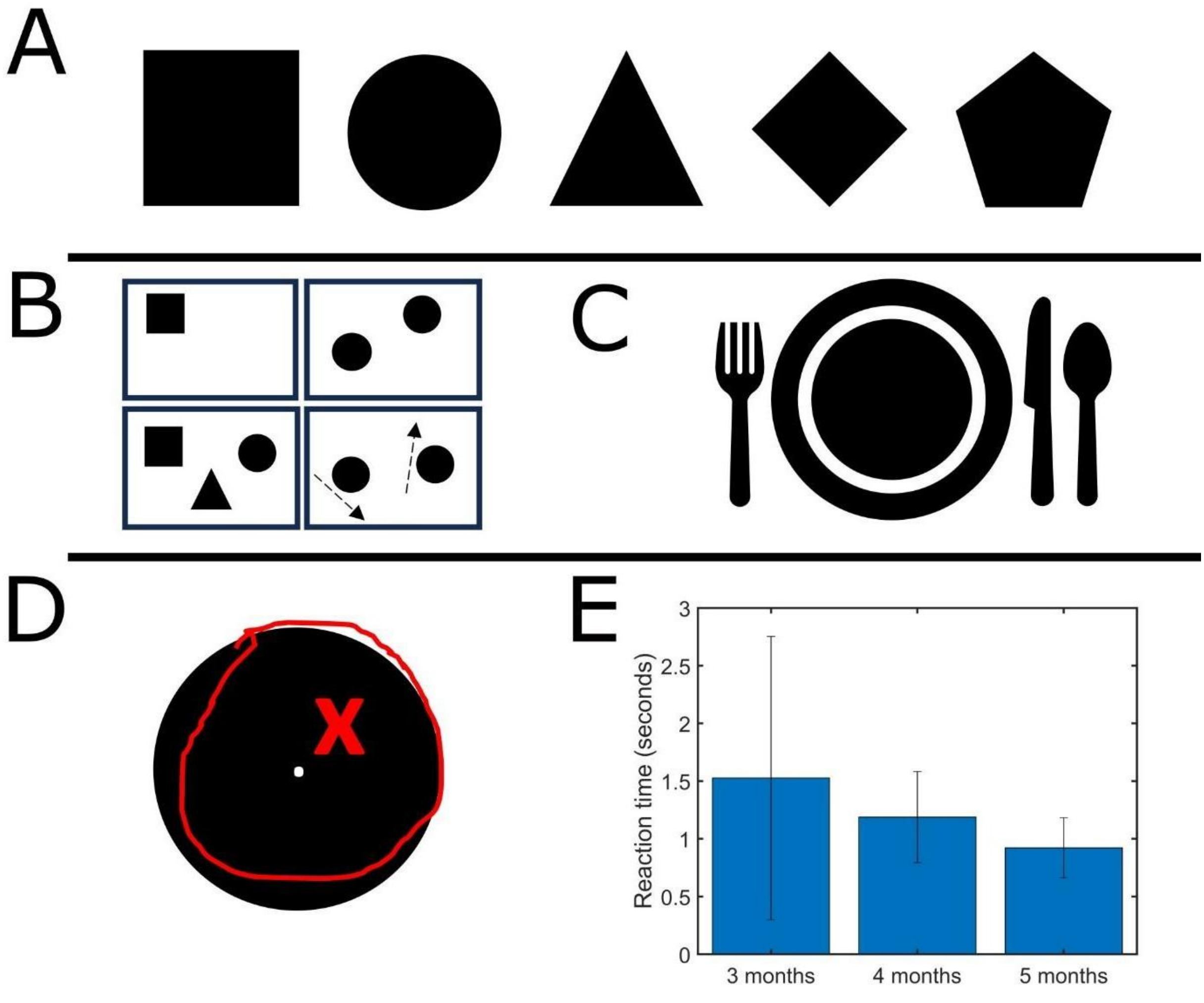

Supplementary Figure 1. Stimuli presented on the screen for shape recognition task. A) Shapes used with a task to name the shape, point to its centre and track the edges of the shape with finger. B) Examples of shapes presented in a computer screen either in isolation or in groups of two or three with the same task. In a different task, tracking of two circular shapes was required. C) High contrast dinner table set presented on the computer screen for visual identification. D) A common error in pointing to the centre (white dot) slightly above to the right (red cross) and missing some part of the left lower part of the objects (red line). E) Reaction time evolution in the left eye for letter identification task. Note that at 3- and 4-months post- surgery (i.e., from the start of study participation), the task was not timed, whereas at 5 months post-surgery the letters were presented for 500 ms.

Activities of Daily Living (ADL)

Sock sorting

We performed a sock sorting task that was inspired by retinal visual implant recipients [6]. The subject was comfortably seated at the table on which there was a bowl containing 8 socks, indistinguishable by touch, in white, black, and grey colours. The task was to sort the socks into 3 piles of different colours and identify which pile is which. After 3 trials, the task was made more complex by increasing the total amount of socks to 30. Results were recorded as the number of correctly sorted socks as a function of time (Supplementary Figure 2A). We performed one task during every month of implantation, and one repetition 6 months after the implant was removed.

Tabletop task

During the tabletop task, the participant was asked to visually search for various everyday objects placed on a table, identify each item by name, and then reach out to grasp it. The task was performed using vision alone, without the aid of tactile exploration prior to reaching. Lighting conditions were kept consistent across sessions, while the colour of the tablecloth was varied to avoid over-standardisation of the visual context. The main challenge observed during this task was impaired hand–eye coordination, likely related to reduced depth perception.

To further investigate spatial localisation and mitigate the depth-related challenge, we developed a second tabletop task using a black board (45 × 64 cm) divided into four equal zones, arranged from nearest (zone 1) to farthest (zone 4) relative to the participant. In each trial, up to eight white stickers were randomly placed across the zones, with both their number and location varied between trials. The participant's task was to visually locate all the stickers and place a pawn on each one. No time constraints were imposed. Performance was quantified as the number of correctly identified sticker positions (Supplementary Figure 2B).

Tic-Tac-Toe

We adapted the traditional Tic-Tac-Toe game into a large-scale, accessible format. We used a black board (53 x 63 cm), with a tactile edge and white lines (2 cm width), as well as the pawns of 'O' (circle) and 'X' (cross) shapes, with diameters of 6 cm and 9 cm, respectively, and a height of 1 cm. This enlargement was intended to accommodate visual input and allow the tactile exploration. The activity was structured as a two-player game, maintaining the original rules of Tic-Tac-Toe. The modified game served both as an investigative tool and as an engaging, game-based activity to increase participant motivation and natural interaction. Spatial skills and memory strategies could be observed in within an entertaining context.

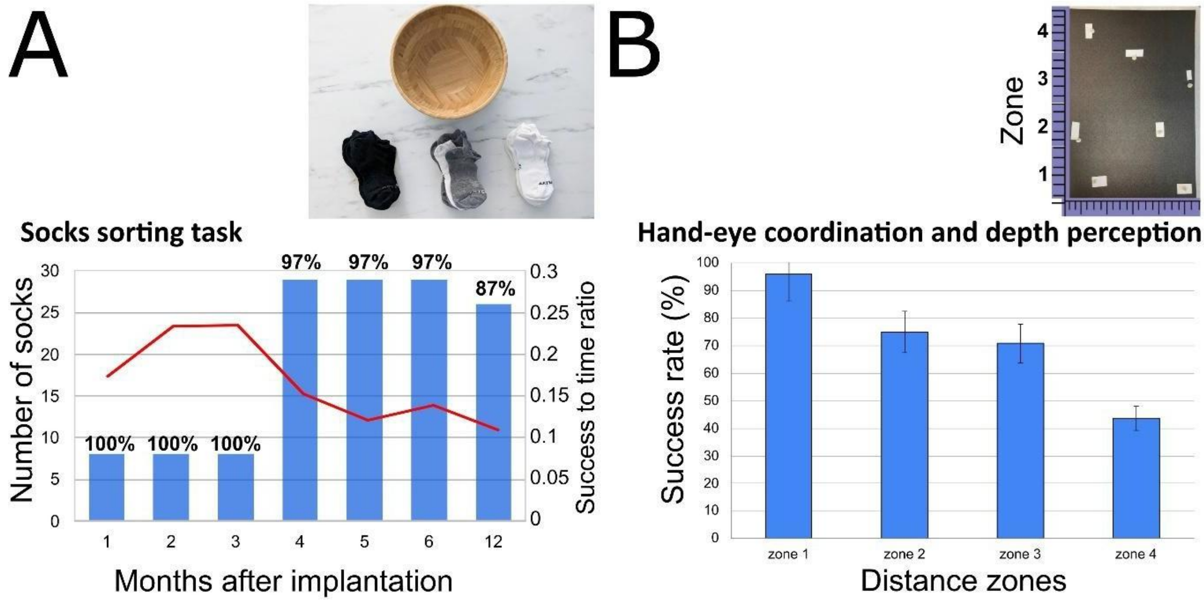

Supplementary Figure 2. Illustrations and results of Activities of Daily Living tasks. A) The sock sorting task was performed once during each month of implantation and 6 months after the implant had been removed. The percentages above the bars indicate the correctness of the answers. B) Hand-eye coordination and depth perception task.

Orientation and Mobility (O&M)

We trained and assessed Orientation and Mobility (O&M) skills using Nemty®, a Virtual Reality (VR) environment consisting of a treadmill and three large screens positioned at eye-level. The set-up was designed to simulate real-world navigation challenges, while ensuring safety through the use of support bars (Supplementary Figure 3A). Similar VR training has shown to have a positive effect on real-world navigation tasks in patients [3,4]. In a subsequent experiment, we used an environment composed of a corridor scene with various high-contrast static and moving obstacles (tall cubic boxes) located either in the left, right or middle of the path. The participant was instructed to walk from one end of the corridor to the other while avoiding these obstacles. The task relied solely on visual cues—no tactile or auditory information was provided—to evaluate how the participant used vision to guide movement and avoid collisions. Ambient lighting was kept constant across sessions. Each trial ended when the participant reached the end of the track (approximately 3 minutes).

Following this virtual training, a real-world orientation and mobility experiment was conducted in a 22 x 2m indoor corridor (Supplementary Figure 3B), similar to a previously published method [5]. Cardboard boxes (180 × 70 × 60 cm) were placed along the corridor at 2-meter intervals with position noise, with a total of 10 obstacles used. The participant's task was to walk the full length of the corridor while visually detecting and avoiding the obstacles, again using vision alone, without physical probing or guidance.

We recorded the completion time and number of obstacle collisions (defined as unwanted contacts with obstacles) for each trial. We measured the average preferred walking speed

(PPW) of the patient walking in a straight empty corridor with a cane. For each obstacle trial (*i*), we calculated percentage of preferred walking speed (PPWS) as:

$$PPWS_i = \frac{v_i}{\text{PPW}} \times 100$$

where v_i is the patient's mean walking speed during trial *i.*

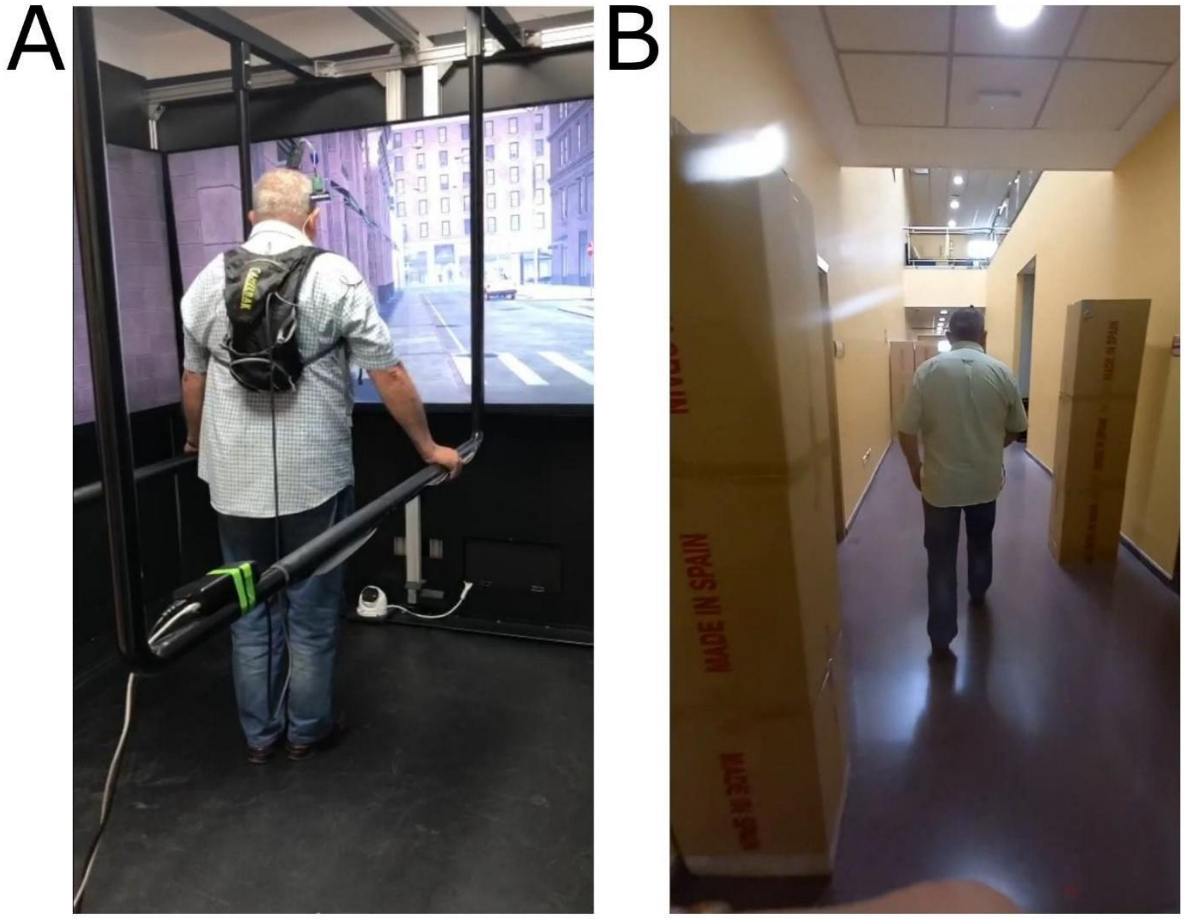

Supplementary Figure 3. Orientation and mobility training and assessment settings. A) Virtual corridor designed to train obstacle avoidance using high-contrast boxes while patient was walking on a treadmill. B) Real corridor experiment where the participant was walking along a 22-meter indoor corridor while avoiding large cardboard boxes spaced at variable distances minimally 2 meters apart.

Statistical analysis

Trial-wise PPWS was modelled as a linear trend using Prais–Winsten generalised least squares to account for AR(1) autocorrelation. 95% confidence intervals for the slope and fitted trend were obtained via a moving-block bootstrap with a block length of 3. Residuals exhibited mild negative first-order autocorrelation (AR(1) $\rho = -0.39$), indicating alternating trial-to-trial deviations around the fitted trend; the Prais–Winsten model and residual block bootstrap accounted for this dependence. Collision counts were summarised with a Theil–Sen robust trend estimate; 95% CIs and confidence bands were derived via moving-block bootstrap (block length = 3).

Results

As shown in Supplementary Figure 4A–B, across 10 trials PPWS increased over time (Prais–Winsten GLS; slope= 1.27 %PPWS per trial, 95% CI 0.91–1.58; AR(1) ρ = −0.39), while collisions decreased (Theil– Sen slope = −1.00 collisions per trial, 95% CI −1.20 to −0.80). As a descriptive summary, the median PPWS in the last three trials exceeded the first three by +10.25 percentage points, and collisions were 7 fewer. Together, these trends indicate that—although obstacles initially constrained pace—the participant progressively walked faster, approaching their baseline preferred speed, and showed a clear learning curve.

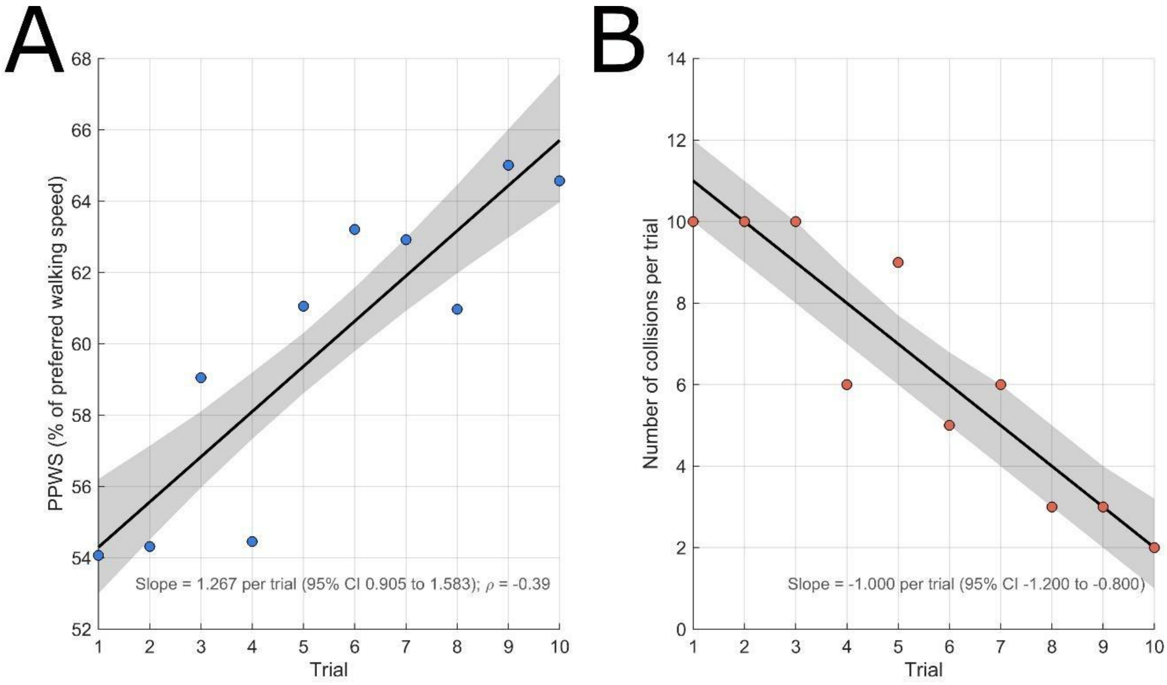

Supplementary Figure 4. Within-subject time course of walking performance in Orientation and Mobility corridor tasks across 10 trials. A) PPWS (percentage of preferred walking speed) increased over time. The solid line is the Prais–Winsten GLS trend (AR(1) errors), and the grey band is the 95% confidence band from a residual moving-block bootstrap (block length = 3). Estimated slope = +1.27 %PPWS per trial (95% CI 0.905–1.583); residual autocorrelation ρ =−0.39. B) Collisions per trial decreased over time. The solid line is the Theil–Sen robust trend; the grey band is the 95% confidence band from a residual moving-block bootstrap (block length = 3). Estimated slope = −1.00 collisions per trial (95% CI −1.20 to −0.80).

Summary

Here, we implemented a visual-rehabilitation protocol that gradually increased task complexity to sustain and improve the patient's everyday visual performance. The protocol started with high-contrast, screen-based tasks which provided a foundation for progressively more complex activities. Initially, the patient practiced basic shape and letter recognition under controlled conditions. Visual skills were then transferred to real-world application—such as sock sorting, tabletop object search, and an adapted Tic-Tac-Toe. Finally, orientation and mobility experiments in both virtual and physical corridors confirmed that visual cues alone can guide locomotion more safely and efficiently with practice.

The visual perceptual training used in this study was intended as a complementary component and was not designed to systematically measure task-specific improvements. While the effects of rehabilitation may be partially reflected in the observed improvements in visual acuity and electrophysiological responses, it remains unclear to what extent the training itself contributed to the recovery of vision. Nonetheless, the visual tasks enhanced the participant’s engagement and motivation. The participant also reported continuing similar activities at home and occasionally experiencing functional visual improvements, such as recognising clothing worn by family members or perceiving distant landscapes. Overall, the incorporation of visual tasks appeared to support sustained participation and engagement throughout the study.

Supplementary Statistical Analyses

Component	Eye	Session	t(df)	p_{raw}	p_{FDR}	d	%Δ	sig_{FDR}
**N1**	Left	Before	0.37(4)	0.729	0.761	0.41	1.5%	no
**N1**	Left	2 m	-0.49(4)	0.647	0.706	-0.54	-2.0%	no
**N1**	Left	5 m	3.59(4)	0.023	0.037	3.93	14.6%	yes
**N1**	Left	6 m f/u	4.82(4)	0.009	0.019	5.28	19.6%	yes
**N1**	Right	Before	6.55(4)	0.003	0.008	7.18	26.6%	yes
**N1**	Right	2 m	-3.09(4)	0.037	0.049	-3.39	-12.6%	yes
**N1**	Right	5 m	3.83(4)	0.019	0.032	4.20	15.6%	yes
**N1**	Right	6 m f/u	3.34(4)	0.029	0.042	3.66	13.6%	yes
**P1**	Left	Before	2.28(4)	0.085	0.102	2.49	9.6%	no
**P1**	Left	2 m	21.21(4)	0.000	0.001	23.23	89.7%	yes
**P1**	Left	5 m	17.29(4)	0.000	0.001	18.94	73.1%	yes
**P1**	Left	6 m f/u	10.58(4)	0.000	0.003	11.59	44.7%	yes
**P1**	Right	Before	7.31(4)	0.002	0.006	8.01	30.9%	yes
**P1**	Right	2 m	-0.15(4)	0.889	0.889	-0.16	-0.6%	no
**P1**	Right	5 m	5.26(4)	0.006	0.015	5.76	22.2%	yes
**P1**	Right	6 m f/u	9.18(4)	0.001	0.004	10.05	38.8%	yes
**N2**	Left	Before	0.74(4)	0.500	0.571	0.81	7.1%	no
**N2**	Left	2 m	10.62(4)	0.000	0.003	11.63	101.0%	yes
**N2**	Left	5 m	7.74(4)	0.001	0.006	8.48	73.7%	yes
**N2**	Left	6 m f/u	5.50(4)	0.005	0.014	6.03	52.4%	yes
**N2**	Right	Before	2.72(4)	0.053	0.067	2.98	25.9%	no
**N2**	Right	2 m	4.19(4)	0.014	0.028	4.59	39.9%	yes
**N2**	Right	5 m	3.30(4)	0.030	0.042	3.61	31.4%	yes
**N2**	Right	6 m f/u	3.94(4)	0.017	0.031	4.31	37.5%	yes

Supplementary Table 1. Latencies of patient VEP components (N1, P1, N2) across testing sessions compared to a control group (n = 5) using Crawford & Howell's modified t-test [10]. Raw (p_{raw}) and Benjamini–Hochberg FDR–adjusted (p_{FDR}) p-values are given alongside Cohen's d (d) and percentage difference (Δ %). The final column denotes whether the FDR-adjusted p-value meets the significance threshold (p_{FDR} < 0.05; "yes"/"no"), and rows shaded light grey indicate significant differences. We conducted four sessions: pre-surgery ("Before"), two months ("2 months") and at five months ("5 months") after the start of the clinical trial (trial ended at 6 months), and six-month follow-up ("6-month f-u").

Component	Eye	Session	t(df)	p_{raw}	p_{FDR}	d	Δ(%)	sig_{FDR}
**P1**	Left	Before	–5.16(4)	0.003	0.026	–5.65	–67.5 %	yes
**P1**	Left	2 m	–1.44(4)	0.112	0.223	–1.58	–18.8 %	no
**P1**	Left	5 m	–2.56(4)	0.031	0.084	–2.80	–33.4 %	no
**P1**	Left	6 m f/u	–2.35(4)	0.039	0.090	–2.58	–30.7 %	no
**P1**	Right	Before	–5.95(4)	0.002	0.026	–6.52	–77.8%	yes
**P1**	Right	2 m	–3.94(4)	0.008	0.027	–4.32	–51.5%	yes
**P1**	Right	5 m	–4.49(4)	0.005	0.026	–4.92	–58.6%	yes
**P1**	Right	6 m f/u	–4.26(4)	0.007	0.026	–4.67	–55.7%	yes
**N2**	Left	Before	–1.66(4)	0.172	0.299	–1.82	–78.9 %	no
**N2**	Left	2 m	1.59(4)	0.187	0.299	1.74	75.4%	no
**N2**	Left	5 m	0.61(4)	0.576	0.576	0.67	28.8%	no
**N2**	Left	6 m f/u	1.10(4)	0.331	0.353	1.21	52.4%	no
**N2**	Right	Before	–1.44(4)	0.223	0.324	–1.58	–68.3 %	no
**N2**	Right	2 m	–1.16(4)	0.310	0.353	–1.27	–55.1 %	no
**N2**	Right	5 m	–1.37(4)	0.243	0.324	–1.50	–64.9 %	no
**N2**	Right	6 m f/u	–1.17(4)	0.309	0.353	–1.28	–55.3 %	no

Supplementary Table 2. Amplitudes of patient VEP components P1 (P1-N1) and N2 (P1-N2) across testing sessions (pre-surgery = Before; two months = 2 months; five months = “5 months; six-month follow-up = 6-month f-u) compared to a control group (n = 5) using Crawford & Howell’s modified t-test [10]. Raw (p_{raw}) and Benjamini–Hochberg FDR– adjusted (p_{FDR}) p-values are given alongside Cohen’s d (d) and percentage difference (Δ %). The final column denotes whether the FDR-adjusted p-value meets the significance threshold (p_{FDR}< 0.05; “yes”/“no”), and rows shaded light grey indicate significant differences.

Eye	Session	t(df)	p_{raw}	p_{FDR}	CI_{lower}	CI_{upper}	sig_{FDR}
**Left**	Before vs 2 m	2.92(2)	0.099	0.15	-0.28	0.05	no
**Left**	Before vs 5 m	5.75(2)	0.029	0.11	-0.31	-0.04	no
**Left**	Before vs 6 m f/u	2.83(2)	0.106	0.15	-0.28	0.06	no
**Right**	Before vs 2 m	5.08(2)	0.037	0.11	-0.05	-0.004	no
**Right**	Before vs 5 m	1.98(2)	0.186	0.22	-0.15	0.06	no
**Right**	Before vs 6 m f/u	0.48(2)	0.679	0.68	-0.11	0.08	no

Supplementary Table 3. Steady-state visual evoked potential (SSVEP) amplitude averaged across the three channels with the highest response (Oz, O2, CB2) in the patient's left and right eyes. All sessions (pre-surgery = Before; two months = 2 months; five months = "5 months; six-month follow-up = 6-month f-u) were compared to baseline ("Before") using paired-samples t-tests. Raw (p_{raw}) and Benjamini–Hochberg FDR–adjusted (p_{FDR}) p-values are given alongside lower and upper confidence intervals (CI). The final column denotes whether the FDR-adjusted p-value meets the significance threshold (p_{FDR} < 0.05; "yes"/"no").

Eye	Session	t(df)	p_{raw}	p_{FDR}	d	Δ(%)	sig_{FDR}
**Left**	Before	–3.04	0.019	0.039	–3.33	–93.2%	yes
**Left**	2 m	–2.05	0.055	0.072	–2.25	–62.8%	no
**Left**	5 m	–1.59	0.093	0.093	–1.74	–48.8%	no
**Left**	6 m f/u	–1.93	0.063	0.072	–2.11	–59.1%	no
**Right**	Before	–3.14	0.017	0.039	–3.44	–96.3%	yes
**Right**	2 m	–2.91	0.022	0.039	–3.19	–89.1%	yes
**Right**	5 m	–2.80	0.024	0.039	–3.07	–85.9%	yes
**Right**	6 m f/u	–2.81	0.024	0.039	–3.08	–86.2%	yes

Supplementary Table 4. Crawford–Howell t-tests [10] comparing the patient's SSVEP amplitude at Oz in each session (pre-surgery = Before; two months = 2 months; five months = "5 months; six-month follow-up = 6-month f-u) to a control group (n = 5). Raw (p_{raw}) and Benjamini–Hochberg FDR–adjusted (p_{FDR}) p-values are given alongside Cohen's d (d) and percentage difference (Δ %). The final column indicates whether the FDR-adjusted p-value meets the significance threshold (p_{FDR}< 0.05; "yes"/"no"), and rows shaded light grey denote significant deviations from the control mean.

Individual Responses of Control Participants

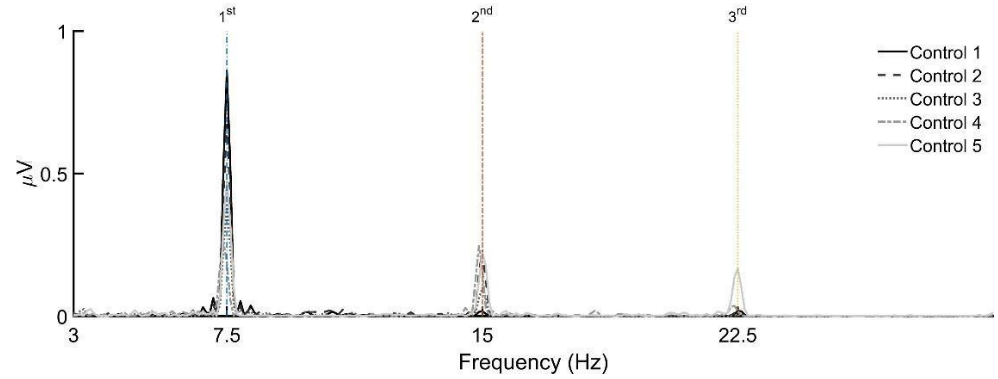

Supplementary Figure 5. Steady-state visual evoked potentials in controls (n = 5). Grand-average amplitude spectra over Oz, POz, O1, and O2 (four highest-response occipital channels), plotted per participant, with peaks at the fundamental (7.5 Hz) and second and third harmonics (15 and 22.5 Hz). Same data as shown in Figure 4A (main text), plotted separately for each control participant.

References

1. Srivastava NR, Troyk PR, Dagnelie G. Detection, eye–hand coordination and virtual mobility performance in simulated vision for a cortical visual prosthesis device. *J Neural Eng.* 2009;6(3):035008. doi:10.1088/1741-2560/6/3/035008

2. Chen SC, Suaning GJ, Morley JW, Lovell NH. Rehabilitation regimes based upon psychophysical studies of prosthetic vision. *J Neural Eng.* 2009;6(3):035009. doi:10.1088/1741-2560/6/3/035009

3. Kim A, Schweighofer N, Finley JM. Locomotor skill acquisition in virtual reality shows sustained transfer to the real world. *J NeuroEngineering Rehabil.* 2019;16:1–10. doi:10.1186/s12984-019-0584-y

4. Neugebauer A, Sipatchin A, Stingl K, Ivanov I, Wahl S. Influence of open-source virtual-reality based gaze training on navigation performance in Retinitis pigmentosa patients in a crossover randomized controlled trial. *Plos one.* 2024;19(2):e0291902. doi:10.1371/journal.pone.0291902

5. van Steveninck JdR, van Gestel T, Koenders P, et al. Real-world indoor mobility with simulated prosthetic vision: The benefits and feasibility of contour-based scene simplification at different phosphene resolutions. *Journal of Vision.* 2022;22(2):1. doi:10.1167/jov.22.2.1

6. Dagnelie G, Christopher P, Arditi A, et al. Performance of real-world functional vision tasks by blind subjects improves after implantation with the Argus® II retinal prosthesis system. *Clin Experiment Ophthalmol.* 2017;45(2):152–159. doi:10.1111/ceo.12812

7. Geruschat DR, Flax M, Tanna N, et al. FLORA™: Phase I development of a functional vision assessment for prosthetic vision users. *Clin Experiment Ophthalmol.* 2015;98(4):342–347. doi:https://doi.org/10.1111/cxo.12242

8. Finger RP, McSweeney SC, Deverell L, et al. Developing an instrumental activities of daily living tool as part of the low vision assessment of daily activities protocol. *Invest Ophthalmol Vis Sci.* 2014;55(12):8458–8466. doi:10.1167/iovs.14-14732

9. Kotecha A, Zhong J, Stewart D, da Cruz L. The Argus II prosthesis facilitates reaching and grasping tasks: a case series. *BMC ophthalmology.* 2014;14(1):71. doi:10.1186/1471-2415-14- 71

10. Crawford JR, Howell DC. Comparing an individual's test score against norms derived from small samples. *Clin Neuropsychol.* 1998;12(4):482–486. doi:10.1076/clin.12.4.482.7241
